# Rapid metabolism of exogenous angiotensin II by catecholaminergic neuronal cells in culture media

**DOI:** 10.14814/phy2.12287

**Published:** 2015-02-03

**Authors:** Urmi Basu, Javier Seravalli, Nandakumar Madayiputhiya, Jiri Adamec, Adam J Case, Matthew C Zimmerman

**Affiliations:** 1 Department of Cellular and Integrative Physiology, University of Nebraska Medical CenterOmaha, Nebraska; 2 Department of Biochemistry, Redox Biology Center, University of Nebraska-LincolnLincoln, Nebraska

**Keywords:** Angiotensin II, angiotensin peptides, CATH.a neurons, liquid chromatography-tandem mass spectrometry, neuronal cell culture models

## Abstract

Angiotensin II (AngII) acts on central neurons to increase neuronal firing and induce sympathoexcitation, which contribute to the pathogenesis of cardiovascular diseases including hypertension and heart failure. Numerous studies have examined the precise AngII-induced intraneuronal signaling mechanism in an attempt to identify new therapeutic targets for these diseases. Considering the technical challenges in studying specific intraneuronal signaling pathways in vivo, especially in the cardiovascular control brain regions, most studies have relied on neuronal cell culture models. However, there are numerous limitations in using cell culture models to study AngII intraneuronal signaling, including the lack of evidence indicating the stability of AngII in culture media. Herein, we tested the hypothesis that exogenous AngII is rapidly metabolized in neuronal cell culture media. Using liquid chromatography-tandem mass spectrometry, we measured levels of AngII and its metabolites, Ang III, Ang IV, and Ang-1-7, in neuronal cell culture media after administration of exogenous AngII (100 nmol/L) to a neuronal cell culture model (CATH.a neurons). AngII levels rapidly declined in the media, returning to near baseline levels within 3 h of administration. Additionally, levels of Ang III and Ang-1-7 acutely increased, while levels of Ang IV remained unchanged. Replenishing the media with exogenous AngII every 3 h for 24 h resulted in a consistent and significant increase in AngII levels for the duration of the treatment period. These data indicate that AngII is rapidly metabolized in neuronal cell culture media, and replenishing the media at least every 3 h is needed to sustain chronically elevated levels.

## Introduction

Dysregulation of angiotensin II (AngII)-dependent neuronal signaling in the central nervous system is involved in the pathogenesis of cardiovascular diseases, such as hypertension and chronic heart failure (Phillips and Sumners 1998; Veerasingham and Raizada 2003; Guyenet 2006; Zucker 2006). Activation of the renin–angiotensin system and elevated levels of AngII in the brain are associated with the development and maintenance of various forms of experimental and genetic models of hypertension (Basso et al. 1981; Ganten et al. 1983; Davisson et al. 1998; Morimoto et al. 2001). By acting on specific brain regions that are important in autonomic control of cardiovascular function, including the subfornical organ (SFO), paraventricular nucleus (PVN), and rostral ventrolateral medulla (RVLM), AngII increases neuronal firing leading to the deleterious sympathoexcitation commonly associated with neurocardiovascular diseases (Bains et al. 1992; Ferguson and Bains 1997; Gao et al. 2005; Feng et al. 2008). In addition, AngII stimulation of its type I receptor (AT_1_R) in these cardiovascular control brain regions and others contributes to vasopressin secretion, thirst and salt appetite, and baroreflex modulation (Matsukawa et al. 1991; Reid 1992; Johnson and Thunhorst 1997). AngII can also activate its type 2 receptor (AT_2_R) on central neurons, which often results in opposing responses compared to AT_1_R activation, reflecting the different physiological and pathophysiological roles of AngII mediated via these receptors (Sumners et al. 1994).

Considering the importance of AngII intraneuronal signaling in the pathogenesis of neurocardiovascular diseases, including hypertension and heart failure, numerous studies have been devoted to better understand the precise signaling pathways involved in an attempt to identify new therapeutic targets for these diseases. However, there are significant technical challenges in studying AngII-induced intraneuronal signaling in vivo in animal models of hypertension and heart failure such as: (1) limited number of neurons within a specific cardiovascular control brain region of interest to perform a particular assay; (2) separating intraneuronal signaling events from other pathways activated in neighboring cells including glia and endothelial cells; and (3) separating the signaling mechanisms of other peptides and hormones associated with these diseases from those directly induced by AngII. To circumvent these challenges, neuronal cell culture models have been frequently utilized to examine the specific intraneuronal signaling pathways induced by AngII. Such studies have clearly identified important roles for calcium, reactive oxygen species, kinases, and transcription factors in the AngII intraneuronal signaling pathway (Sun et al. 2003; Zimmerman et al. 2004, 2005; Haack et al. 2013).

Nevertheless, there are also limitations in using neuronal cell culture models to recapitulate the AngII intraneuronal signaling events occurring in vivo such as: (1) neuronal cells are often immortalized and/or isolated from a tumor; (2) cells are usually cultured in a hyperoxic environment (i.e., 21% oxygen) as compared to their in vivo environment (1–4% oxygen); and (3) the lack of neighboring glia and endothelial cells may alter a particular response in the cultured neurons that would normally occur in vivo. A fourth limitation in using neuronal cell culture models as it specifically relates to understanding AngII intraneuronal signaling is the lack of evidence indicating the stability of exogenous AngII in neuronal cell culture media. Many studies have examined intra-neuronal responses, such as changes in mRNA levels and protein expression, 1–48 h after a single administration of exogenous AngII into the neuronal cell culture media (Liu et al. 2006; Mitra et al. 2010; Xia et al. 2011). Data from these studies are often interpreted to indicate that the observed changes are also occurring in neurons of various hypertensive and heart failure models in which circulating and/or brain levels of AngII are chronically elevated (van de Wal et al. 2006; Xia et al. 2013). However, it remains unclear if a single treatment of exogenous AngII given to neurons in culture results in a chronic elevation of AngII levels in the media.

In the current study, we tested the hypothesis that exogenous AngII is rapidly metabolized in neuronal cell culture medium and thus fails to remain chronically elevated following a single exogenous administration. We utilized a mouse catecholaminergic neuronal cell line, CATH.a neurons, which express both AT_1_R and AT_2_R. CATH.a neurons have commonly been used by our group and others to study AngII intraneuronal signaling pathways (Sun et al. 2003; Gao et al. 2010; Case et al. 2013). Previous studies have shown that deletion of the AT_1_R from catecholaminergic neurons delays the onset of AngII-dependent hypertension and also reduces the maximal blood pressure response (Jancovski et al. 2013). In addition, catecholaminergic neurons, such as C1 neurons in the RVLM, play an important role in AngII-induced hypertension and blocking the AT_1_R specifically in these neurons can attenuate the AngII-mediated rise in blood pressure (Jancovski et al. 2014). Using liquid chromatography-tandem mass spectrometry, we measured levels of AngII and its metabolites, Ang III, Ang IV, and Ang-1-7, in CATH.a neuronal cell culture media after administration of exogenous AngII. Herein, we report that exogenous AngII is rapidly metabolized in CATH.a neuron media with levels returning to near baseline after 3 h of administration. Further, AngII levels in CATH.a neuron media can be significantly and chronically elevated for at least 24 h with the addition of fresh exogenous AngII every 3 h.

## Materials and Methods

### Neuronal cell culture

Mouse catecholaminergic CATH.a neurons (American Type Culture Collection (ATCC), stock no. CRL–11179) were cultured in RPMI 1640 medium supplemented with 8% normal horse serum (NHS), 4% fetal bovine serum, and 1% penicillin-streptomycin at 37°C with 5% CO_2_ as recommended by ATCC. CATH.a neurons were differentiated for 6–8 days before experimentation by adding *N*^*6*^*,2′-O*-dibutyryladenosine *3′,5′* - cyclic monophosphate sodium salt (1 mmol/L, Sigma-Aldrich, St. Louis, MO) to the culture medium as previously described (Case et al. 2013).

### Liquid chromatography – tandem mass spectrometry (LC-MS/MS)

CATH.a neuronal culture medium was collected after incubation (15 min – 24 h) with AngII (100 nmol/L). The ^13^C- and ^15^N-labeled (Leu, + 7 amu) AngII (H-AngII, Anaspec, Fremont, CA) was added to the media samples at a concentration of 18.2 nmol/L. Proteins were precipitated by addition of 6 volumes of cold neat acetone, typically 50 *μ*L of sample and 300 *μ*L of acetone and stored at −35°C for 1 h. Then, the samples were centrifuged at 4°C for 15 min at 15,000 × *g*. Thereafter, the supernatants were removed and the acetone/water was removed by SpeedVac concentration for 2–3 h at room temperature. The pellet was redissolved into 50 *μ*L of 0.1% formic acid vortexed and centrifuged. The samples were loaded onto V-shaped polyethylene vials (Agilent, Santa Clara, CA) that were previously soaked with 0.1% w/v BSA and dried. The LC-MS/MS method was developed and used for the samples using an Agilent LC1200 HPLC system (Agilent) connected to an ABSciex QTrap4000 (ABSciex, Framingham, MA) operating in the multiple reaction monitoring (MRM) mode with the electrospray operating in the positive mode. Other ion-source conditions were temperature, 500°C, ionization potential, 5500 V, GS1 = 50, GS2 = 25, curtain gas = 30. The transitions monitored and ionization parameters are shown in Table1. Transitions numbered 1,3,5,8 and 9 were used for quantitation while the remaining ones were used for confirmation of peak assignment. Column chromatography was performed via a 2.1 × 50 mm Kinetex C-18 300 Å (Phenomenex, Torrance, CA) at a flow rate of 250 *μ*L/min with a mobile phase gradient from 98% A (0.1% formic acid in LC-water) to 98% B (0.1% formic acid in acetonitrile) over 20 min, with additional holding at 98% B for 2 min and re-equilibration at 98% A for 10 min. Transitions were monitored with an acquisition time of 100 msec/MRM in the nonscheduled mode. The data were analyzed using Analyst Ver 1.4.2. AngII levels were quantified using H-AngII as a standard. The other angiotensin peptides (Ang III, Ang IV, and Ang-1-7) were quantified by comparison to an external calibration curve of the unlabeled commercially available peptides, and H-AngII was used as a surrogate to correct for concentration loss of the peptides during sample preparation. The recoveries of the spiked H-AngII were equal to or higher than 80% for the analytical method. Individual samples were injected in triplicate and the average angiotensin peptide concentrations and standard error are reported. All reagents used for LC-MS/MS analysis were of Mass Spectrometry Grade and all unlabeled angiotensin peptide standards and reagents were purchased from Sigma-Aldrich (St. Louis, MO).

**Table 1:** Instrument parameters for multiple reaction monitoring (MRM)

Transition number	Peptide	Q1 (m/z)	Q3 (m/z)	Declustering potential, V	Collision energy, V
1	AngII	349.6	255.2	40	30
2	AngII	524	784.1	60	30
3	H-AngII	352	255.2	40	30
4	H-AngII	527.5	791.1	60	30
5	AngIII	311.3	256	40	15
6	AngIII	311.3	514	30	12
7	AngIV	388.3	513.6	50	15
8	AngIV	388.3	263.4	45	20
9	Ang1-7	301	371	40	15
10	Ang1-7	301	344	30	15

Transitions 1, 3, 5, 8, and 9 were used for quantitation while transitions 2, 4, 6, 7, and 10 are for confirming peak assignments.

### Statistical analysis

All data are expressed as the mean ± standard error of the mean (SEM) and were analyzed by Student's *t*-test for two-group comparisons or by ANOVA followed by Newman–Keuls correction for multiple comparisons. Statistical analyses were performed using GraphPad Prism 5.0 (La Jolla, CA) statistical and graphical software. Differences were considered significant at *P* < 0.05.

## Results

### Utilizing liquid chromatography – tandem mass spectrometry to detect angiotensin peptides

**Figure 1:**
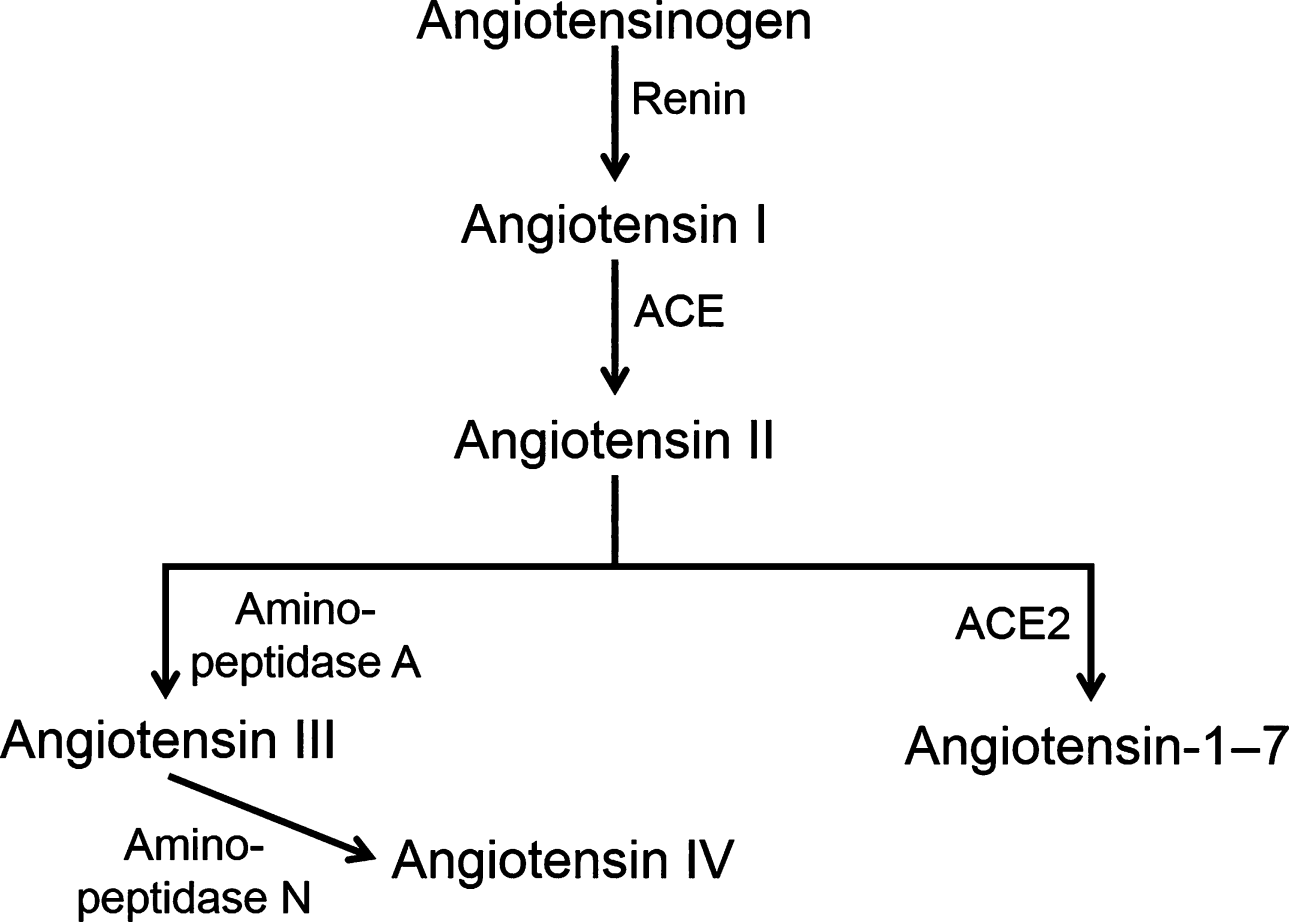
Schematic of the renin–angiotensin system. AngII is generated by angiotensin-converting enzyme (ACE)-induced cleavage of angiotensin I. AngII is subsequently metabolized to Ang III and Ang-1-7 peptide via aminopeptidase A and angiotensin-converting enzyme 2 (ACE2), respectively. Ang III is further metabolized by aminopeptidase N to generate Ang IV.

**Figure 2:**
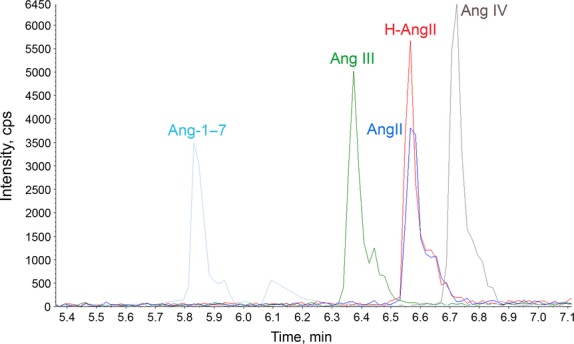
Angiotensin peptides detected by liquid chromatography-tandem mass spectrometry. Representative chromatogram showing the detection of individual angiotensin peptides (AngII, Ang III, Ang IV, and Ang-1-7) in a mixture of the commercially available peptides in a concentration ranging from 12–16 nmol/L. ^13^C- and ^15^N-labeled AngII (H-AngII) was also added to the mixture and used to quantify absolute amounts of the angiotensin peptides in the CATH.a neuronal cell culture media samples analyzed in subsequent experiments.

AngII is generated from angiotensin-converting enzyme (ACE) cleaving angiotensin I, which is produced by renin-induced cleavage of angiotensinogen. Although AngII is considered to be the primary effector peptide of the renin–angiotensin system, AngII can be further metabolized to other angiotensin peptides which have been shown to contribute to cardiovascular function (Campagnole-Santos et al. 1989; Santos et al. 2000; Wilson 2005; Ruiz-Ortega et al. 2007; Marc et al. 2012; Gao et al. 2014). For example, AngII can be cleaved by angiotensin-converting enzyme 2 (ACE2) to form Ang-1-7 or by aminopeptidase A to form Ang III, which is further metabolized by aminopeptidase N to form Ang IV (Fig.1). Although the primary objective of this study was to examine the stability of exogenous AngII in neuronal cell culture media, it was also important to determine if exogenous AngII is metabolized to these other angiotensin peptides. As shown in the representative chromatogram (Fig.2), we were able to successfully separate and detect all four angiotensin peptides from a mixture containing the commercially available peptides ranging in concentration from 12–16 nmol/L. AngII is an octapeptide which under the conditions of the chromatography should accumulate in the +3 charged state due to the positive charges at the terminal amino group as well as the Arg and His side chains. Upon loss of the C-terminus Phe, rendering Ang-1-7, the peptide is much less hydrophobic and thus binds less strongly to the C-18 side chains of the HPLC column particles. Since Phe is more hydrophobic than Asp, removal of Asp instead of Phe from AngII makes the Ang III peptide less hydrophobic than AngII but more than Ang-1-7. Therefore, Ang III elutes between Ang-1-7 and AngII (Fig.2). The hexapeptide Ang IV is generated from the removal of the N-terminus positive side chain Arg from Ang III, while keeping the hydrophobic residues Ile and Phe. As a result of Ang IV being the most hydrophobic, it has the strongest binding and thus appears as the last peak in the chromatogram (Fig.2). To accurately quantify the concentration of these peptides in our subsequent studies using media samples from the CATH.a neuronal culture, we utilized ^13^C- and ^15^N-labeled AngII (H-AngII).

### Exogenous AngII is rapidly metabolized in CATH.a neuronal cell culture media

**Figure 3:**
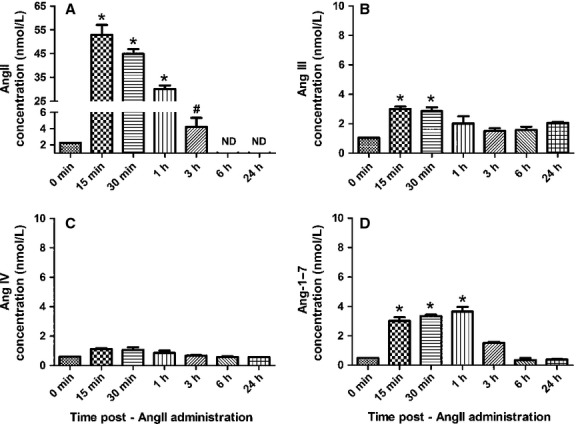
Exogenous AngII levels rapidly decrease within 3 h in CATH.a neuronal cell culture media. Levels of AngII (A), Ang III (B), Ang IV (C), and Ang-1-7 (D) in CATH.a neuronal cell culture media following a single administration of exogenous AngII (100 nmol/L) as measured by liquid chromatography-tandem mass spectrometry. Absolute concentrations were calculated against the standard ^13^C- and ^15^N-labeled AngII peptide. *n* = 3 separate experiments performed in triplicate. *P < 0.05 vs. 0min AngII; ^#^*P* < 0.05 vs. 15 min AngII.

Levels of AngII, Ang III, Ang IV, and Ang-1-7, were measured in CATH.a neuronal cell culture media 15 min – 24 h after a single administration of exogenous AngII (100 nmol/L) into the media. As expected, AngII was significantly elevated in the media 15 min after administration as compared to media collected immediately prior to treatment (Fig.3A). Levels of AngII rapidly decreased over time until 3 h-postadministration when levels of AngII in the media were significantly lower compared to the 15 min time-point and not significantly different than baseline levels. AngII remained low, that is, it was undetectable 6 and 24 h after administration. Interestingly, levels of Ang III (Fig.3B) and Ang-1-7 (Fig.3D) modestly, but significantly, increased 15–60 min after exogenous AngII administration. In contrast, levels of Ang IV remained unchanged compared to baseline levels (Fig.3C). It should be noted that we specifically selected the AngII concentration of 100 nmol/L for these studies because we wanted to replicate, as best we could, the experimental conditions used in other published studies designed to examine the AngII-induced acute and chronic responses in cultured neurons. For example, we previously used 100 nmol/L AngII to study AngII-mediated intraneuronal signaling in CATH.a neurons and have shown that at this concentration AngII-induced Nox4-generated mitochondrial superoxide production contributes to the rapid inhibition of outward K^+^ current (Yin et al. 2010; Case et al. 2013). Furthermore, Sun et al., have reported that 100 nmol/L AngII rapidly inhibits the delayed rectifier K^+^ current and increases neuronal firing rate in primary neurons isolated from the brain (Sun et al. 2002, 2003). Additional studies examining more long-term changes (i.e., hours to days) in the expression of various proteins, including AngII receptors, potassium channels, and transcription factors, also used 100 nmol/L AngII (Gao et al. 2010; Mitra et al. 2010; Haack et al. 2012, 2013).

### Exogenous AngII is stable in neuronal cell culture media in the absence of CATH.a neurons

**Figure 4:**
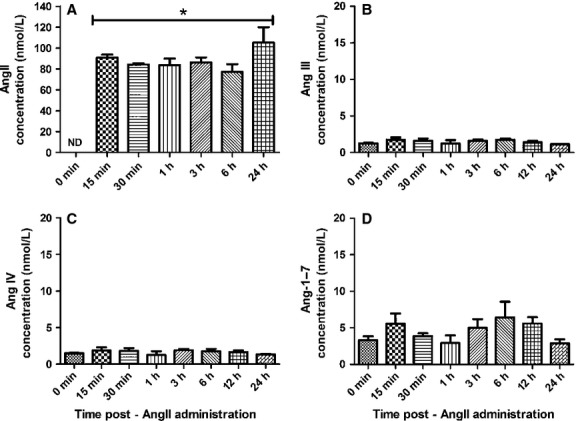
AngII is stable in neuronal cell culture media in the absence of CATH.a neurons. Levels of AngII (A), Ang III (B), Ang IV (C), and Ang-1-7 (D) in cell culture media without CATH.a neurons following a single administration of exogenous AngII (100 nmol/L) as detected by liquid chromatography and tandem mass spectrometry. Absolute concentrations were calculated against the standard ^13^C- and ^15^N-labeled AngII peptide. *n* = 3 separate experiments performed in triplicate. *P < 0.05 vs. 0 min.

The rapid decrease in exogenous AngII levels in media collected from CATH.a neurons may suggest that the peptide is degraded by the media itself. To examine this possibility, AngII (100 nmol/L) was added to the media in the absence of CATH.a neurons and samples were collected 15 min – 24 h later (Fig.4). AngII levels remained significantly elevated at all time-points with concentrations near the starting concentration of 100 nmol/L (Fig.4A). In contrast, levels of Ang III, Ang IV, and Ang-1-7 were not significantly different than baseline levels (Fig.4B–D).

### Replenishing CATH.a neuronal cell culture media every 3 h with fresh exogenous AngII results in chronic elevated levels

**Figure 5:**
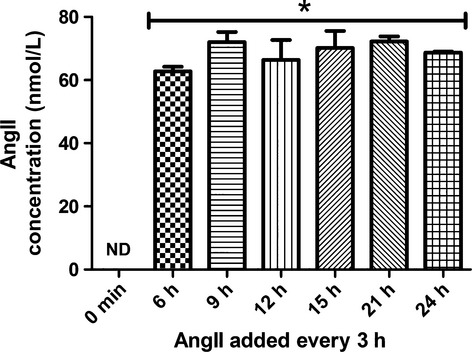
Repeated administration of exogenous AngII every 3 h maintains a chronic elevated level of AngII in CATH.a neuronal cell culture media. Levels of AngII in CATH.a neuronal cell culture media following repeated administration of exogenous AngII (100 nmol/L) into the media every 3 h for 6, 9, 12, 15, 21, and 24 h. Absolute concentrations were calculated against the standard ^13^C- and ^15^N-labeled AngII peptide. *n* = 3 separate experiments performed in triplicate. *P < 0.05 vs. 0 min.

Finally, to determine if levels of AngII could be chronically elevated in CATH.a neuronal cell culture media, we administered fresh exogenous AngII (100 nmol/L) every 3 h and measured AngII for up to 24 h. As shown in Fig.5, AngII levels remained similarly and significantly elevated (63–72 nmol/L) at each time-point compared to baseline levels.

## Discussion

The pathogenesis of various cardiovascular disorders, such as hypertension and chronic heart failure, involves dysregulation of the brain angiotensinergic system (Phillips and Sumners 1998; Veerasingham and Raizada 2003; Zucker 2006). Understanding the precise intraneuronal signaling mechanism(s) driving this dysregulation may lead to the identification of new targets for which novel therapeutics can be developed. As such, numerous studies have focused on examining the intraneuronal signaling molecules and proteins mediating the physiological and pathophysiological responses of AngII (Sumners et al. 2002; Zimmerman 2011; Chan and Chan 2013). Many of these studies have relied on neuronal cell culture models to demonstrate a role for signaling intermediates, such as calcium, reactive oxygen species, kinases, and transcription factors in mediating the AngII-induced response (Sun et al. 2003; Zimmerman et al. 2004, 2005; Haack et al. 2013). However, relating these in vitro observations to in vivo AngII-dependent cardiovascular diseases, such as hypertension and chronic heart failure, where circulating and central levels of AngII are chronically elevated (van de Wal et al. 2006; Xia et al. 2013) is problematic as the stability of exogenously administered AngII in neuronal cell culture media is unknown. In the current study, we examined the stability of exogenous AngII in the culture media of an AngII-sensitive neuronal cell culture model, CATH.a neurons, that has commonly been utilized to study AngII intraneuronal signaling (Sun et al. 2003; Gao et al. 2010; Case et al. 2013). Herein, we report that levels of exogenous AngII are diminished to near baseline levels within 3 h of treatment, and that media should be replenished with fresh AngII every 3 h to maintain chronically elevated levels of the peptide in this neuronal cell culture model.

Acute stimulation (i.e., minutes) of cultured neurons with AngII results in a rapid increase in intracellular calcium (Zimmerman et al. 2005), inhibition of outward potassium current (*I*_Kv_) (Sun et al. 2003; Yin et al. 2010), and an increase in neuronal firing (Sun et al. 2002). Chronic stimulation (i.e., hours to days) of AngII-sensitive cultured neurons leads to alterations in the expression of angiotensin receptors (Liu et al. 2006; Xia et al. 2011), potassium channel proteins (Gao et al. 2010), and transcription factors (Mitra et al. 2010). However, in most, if not all, of these chronic stimulation studies, a single administration of exogenous AngII was given to neurons in culture. This begs the questions of whether AngII remains stable in the culture media for hours or days to continuously activate its receptors to mediate these changes in protein expression or if these changes are a result of the immediate stimulation of AngII receptors followed by prolonged intraneuronal signaling events. With these questions unanswered, it is difficult to compare results obtained from these types of in vitro studies with data obtained from in vivo cardiovascular disease models in which AngII receptors are likely constantly activated due to the chronically elevated levels of AngII. Our new data presented herein indicates that levels of exogenously administered AngII in neuronal cell culture media are decreased to near baseline levels within 3 h; thus, suggesting that changes in protein expression at later time points are due to prolonged intraneuronal signaling events rather than continuous activation of AngII receptors. In fact, we observed that within 15 min of treating cells with 100 nmol/L AngII only 53 ± 4 nmol/L AngII remained in the media, and by 3 h post-treatment, less than 5 nmol/L AngII remained. As we report, some of the exogenous AngII is metabolized in the media to Ang III and Ang-1-7, as the concentration of these two peptides increased 15 min after AngII treatment. We speculate that most of the AngII lost within the first 15 min of treatment is bound to the AngII receptors and internalized via receptor-mediated endocytosis. In support of this hypothesis, previous studies using aortic smooth muscle cells have demonstrated that after AngII binds to its receptor, the complex is internalized with a half-time of less than 2 min (Anderson et al. 1993). In addition, previous reports indicate a functional role for internalized AngII after it has bound to the AT_1_R and the complex has been endocytosed (Harris 1999). For example, the complete AT_1_R-induced activation of mitogen-activated protein kinase (MAPK) is believed to be dependent on internalization (Yang et al. 1997). Furthermore, nuclear membrane-associated angiotensin receptors have been identified (Pendergrass et al. 2006; Gwathmey et al. 2009) and may be stimulated by internalized AngII and/or by AngII generated via an intracellular renin–angiotensin system (Grobe et al. 2008).

After observing the rapid decrease in exogenously administered AngII in the media of our neuronal cell culture model, we questioned whether this was cell-mediated or nonspecific degradation of AngII in the media. To address this question, we measured levels of AngII in culture media in the absence of CATH.a neurons after a single exogenous administration of 100 nmol/L AngII, and found that AngII is relatively stable in the media. More specifically, the concentration of AngII at all time-points measured (15 min – 24 h) was approximately 80 nmol/L or greater. Taken together with our results in the presence of CATH.a neurons, these data suggest that the rapid loss of exogenous AngII in the media of CATH.a neurons is mostly cell-dependent with the AngII being metabolized to Ang III and Ang-1-7, or binding to its receptors, along with some nonspecific degradation of the peptide. As such, it is likely that the stability of exogenous AngII in cultured media will be drastically different between various cells types. That is, the expression levels of aminopeptidase A, which cleaves AngII to Ang III, ACE2, which cleaves AngII to Ang-1-7, and/or AngII receptors in a particular cell type will influence the stability of exogenous AngII in the respective media. Although we did not observe a change in Ang IV in the current study with CATH.a neurons, it is tempting to speculate that the amount of aminopeptidase N, which cleaves Ang III to Ang IV, expressed by a particular cell line will also contribute to the levels of these angiotensin peptides in culture media. Furthermore, it is possible that the stability of AngII in culture media may be experimentally manipulated by the exogenous administration of aminopeptidase inhibitors, ACE2 inhibitors, and/or AngII receptor antagonists.

In conclusion, our results demonstrate that a single exogenous administration of AngII to CATH.a neurons in culture does not result in chronically elevated levels of AngII in the media. As such, we sought to determine if multiple and sequential administration would keep AngII levels elevated chronically. Our data show that giving fresh AngII (100 nmol/L) every 3 h to cultured CATH.a neurons does indeed keep levels of AngII in the media significantly elevated for at least 24 h. We believe cell culture models with chronically elevated levels of AngII are the ideal in vitro models that one should utilize to study the precise AngII-dependent intracellular signaling events that occur in cardiovascular diseases associated with systemic and/or local chronically elevated levels of AngII, including hypertension and chronic heart failure. We posit that keeping levels of AngII chronically elevated in cell culture models will change the cellular response induced by AngII compared to the response induced by a single administration. Alternatively, it is possible that AngII levels may not need to be chronically elevated in cell culture media to induce long-term effects as the initial stimulation of AngII receptors may activate intracellular signaling pathways that lead to chronic changes in the expression of AngII receptors, ion channel proteins, and transcription factors. To address these hypotheses, our laboratory is currently investigating changes in AngII-dependent intracellular signaling events in CATH.a neurons exposed to single versus repeated administration of AngII. More specifically, we are examining changes in AngII receptor expression, levels of reactive oxygen species and antioxidants, intracellular calcium concentration, activation of transcription factors, and expression of ion channels. Importantly, we are comparing any changes in response to single or repeated administration of AngII to those that we observe in cardiovascular control brain nuclei (e.g. SFO, PVN, RVLM) collected from animal models in which AngII is known to be chronically elevated in the brain, including hypertensive and chronic heart failure models. We believe these, and other, future studies will provide further insight into the importance of establishing cell culture models with chronically elevated levels of AngII to improve our understanding of AngII intracellular signaling pathways.
